# Supplementary figures and images for: Depletion of endogenously biotinylated carboxylases enhances the sensitivity of TurboID-mediated proximity labeling in Caenorhabditis elegans
Source: J Biol Chem. 2022 Aug 3;298(9):102343. doi: 10.1016/j.jbc.2022.102343 (PMC9437848; doi:10.1016/j.jbc.2022.102343)

# Figure S1

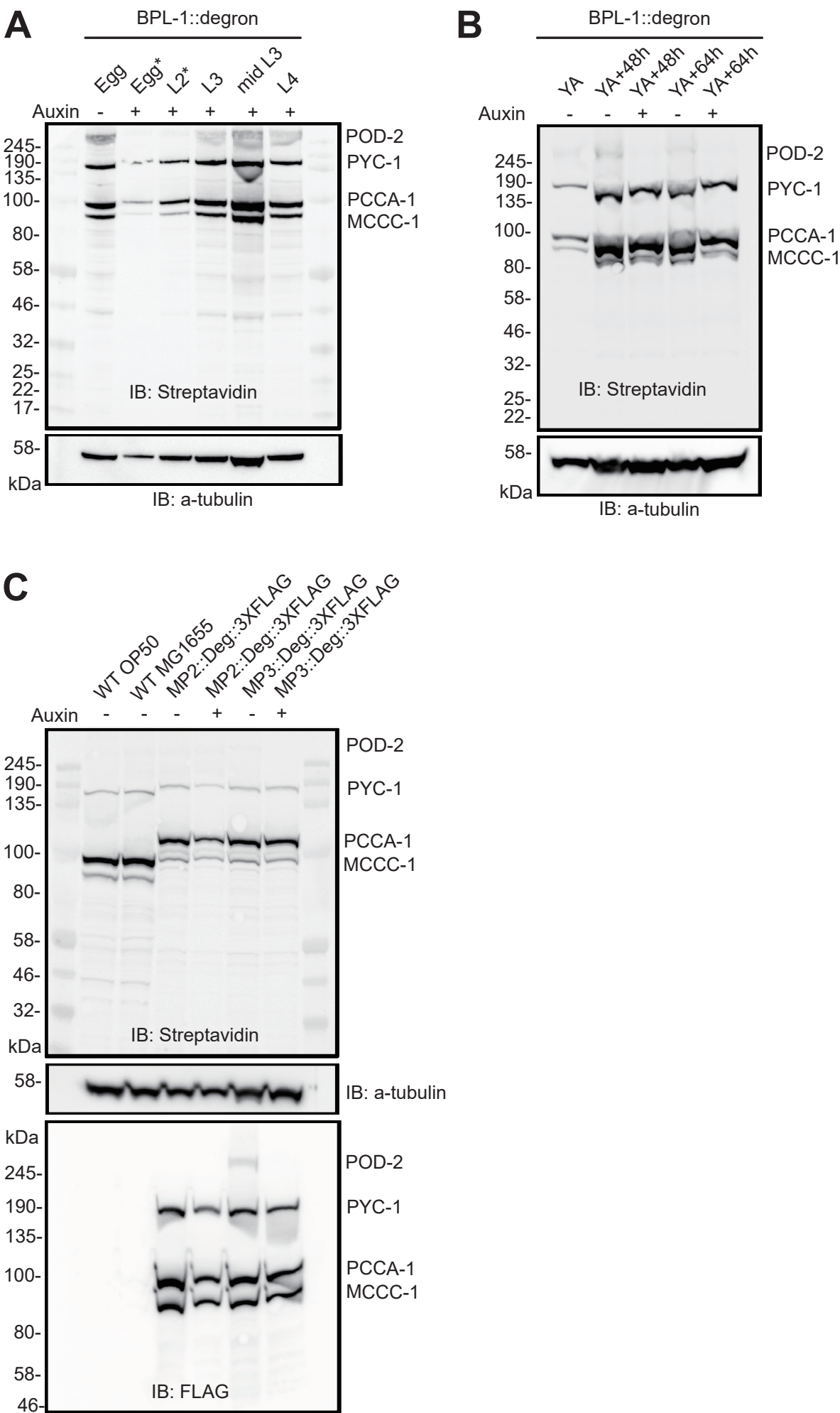

Supplement: Supplemental Figure S1 — AID-mediated depletion of BPL-1 and MP3.A, the effect of AID-mediated knockdown of BPL-1 starting at different developmental stages on biotinylation of MP3 carboxylases in C. elegans. Worms were transferred to auxin plates starting from egg, L2, early or mid-L3 or L4 stages, and harvested as young adults (YA). Please note that the growth of animals in the conditions marked with an asterisk (Egg∗ and L2∗) was arrested. We nevertheless harvested them in their arrested state and processed them for Western blot analyses. B, the effect of AID-mediated knockdown of BPL-1 starting at the young adult stage. Worms were transferred to auxin containing plates as young adults and harvested 48 or 64 h post-YA. C, depletion of MP2::AID or MP3::AID. A 5-h incubation on auxin was sufficient to deplete POD-2 but not MCCC-1, PCCA-1 or PYC-1. [file mmc1.pdf]

Figure S2

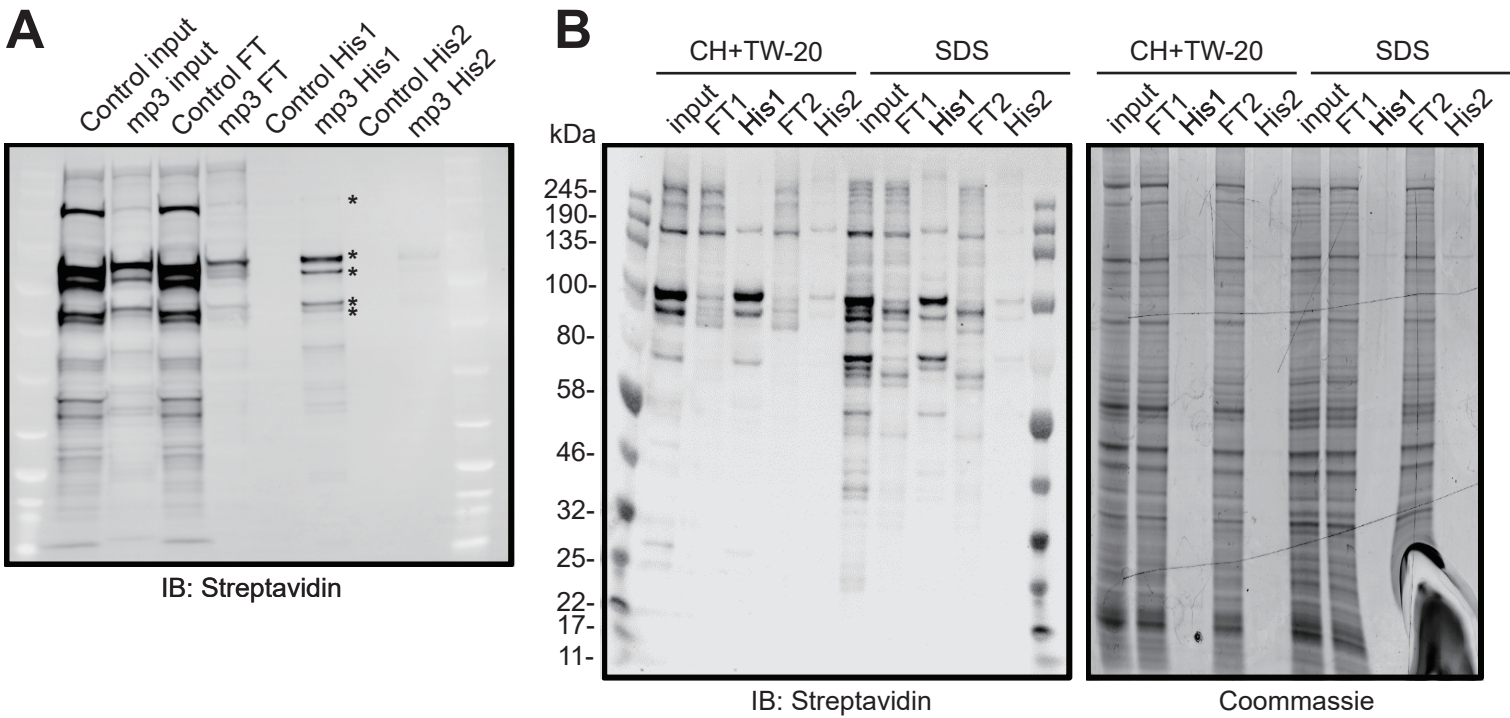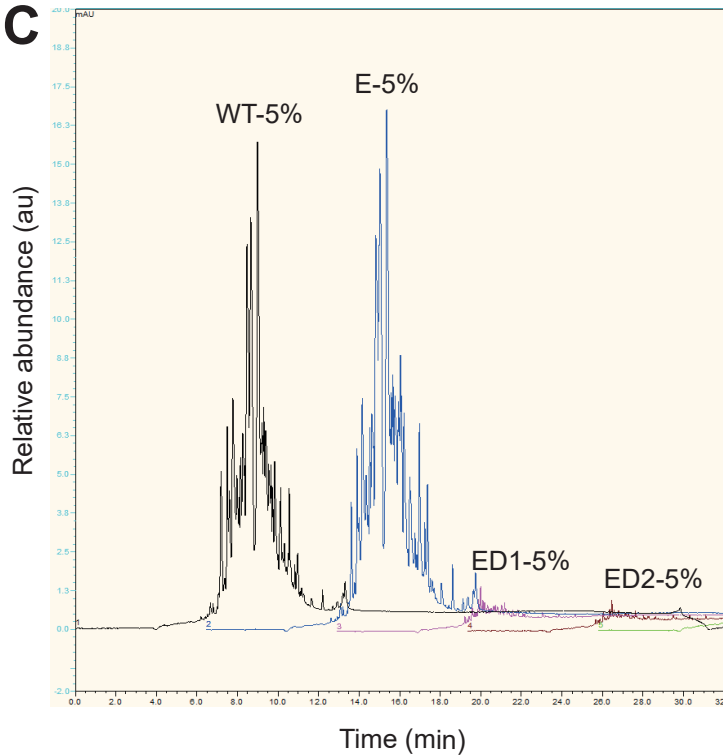

Supplement: Supplemental Figure S2 — Optimizing carboxylase depletion.A, Ni-NTA resin efficiently binds His10-tagged proteins. B, an extraction buffer containing SDS solubilizes the worm proteome more effectively than an extraction buffer containing a mixture of CHAPS, Triton-X100 and Tween-20. C, Ni-NTA depleted and undepleted worm samples following streptavidin purification and on-bead trypsin digestion analysed using an HPLC-UV system to separate peptides. WT: MP3, undepleted; E: ELKS-1::TbID undepleted; ED1: ELKS-1::TbID samples depleted once; ED2: ELKS-1::TbID samples depleted twice. [file mmc2.pdf]
