# Supplementary material for: Depletion of endogenously biotinylated carboxylases enhances the sensitivity of TurboID-mediated proximity labeling in Caenorhabditis elegans
Source: J Biol Chem. 2022 Aug 3;298(9):102343. doi: 10.1016/j.jbc.2022.102343 (PMC9437848; doi:10.1016/j.jbc.2022.102343)

Figure S3

A

Enrichment of synaptic proteins in different samples compared to wild-type worms (log_2 value)

Protein	E-5% vs WT-5%	adj. p-value	ED-50% vs WT-5%	adj. p-value	ED-5% vs WT-5%	adj. p-value
TOM-1	0.12	0.89	3.89	0.00	1.23	0.05
UNC-41	0.93	0.21	4.40	0.00	3.10	0.01
UNC-31	1.40	0.19	4.85	0.00	4.18	0.00
SAD-1	1.30	0.39	4.54	0.01	4.41	0.01
RIC-4	2.51	0.12	5.41	0.00	4.75	0.00
AIPR-1	-0.20	0.84	2.67	0.01	2.67	0.00
GIT-1	0.90	0.20	3.27	0.00	2.79	0.00
CPX-1	4.95	0.00	7.29	0.00	6.74	0.00
C16E9.2	1.49	0.15	3.54	0.00	2.81	0.05
CLA-1	8.72	0.00	10.75	0.00	9.28	0.00
SYD-2	5.33	0.00	7.18	0.00	6.99	0.00
SIPA-1	4.30	0.00	5.92	0.00	6.04	0.00

B

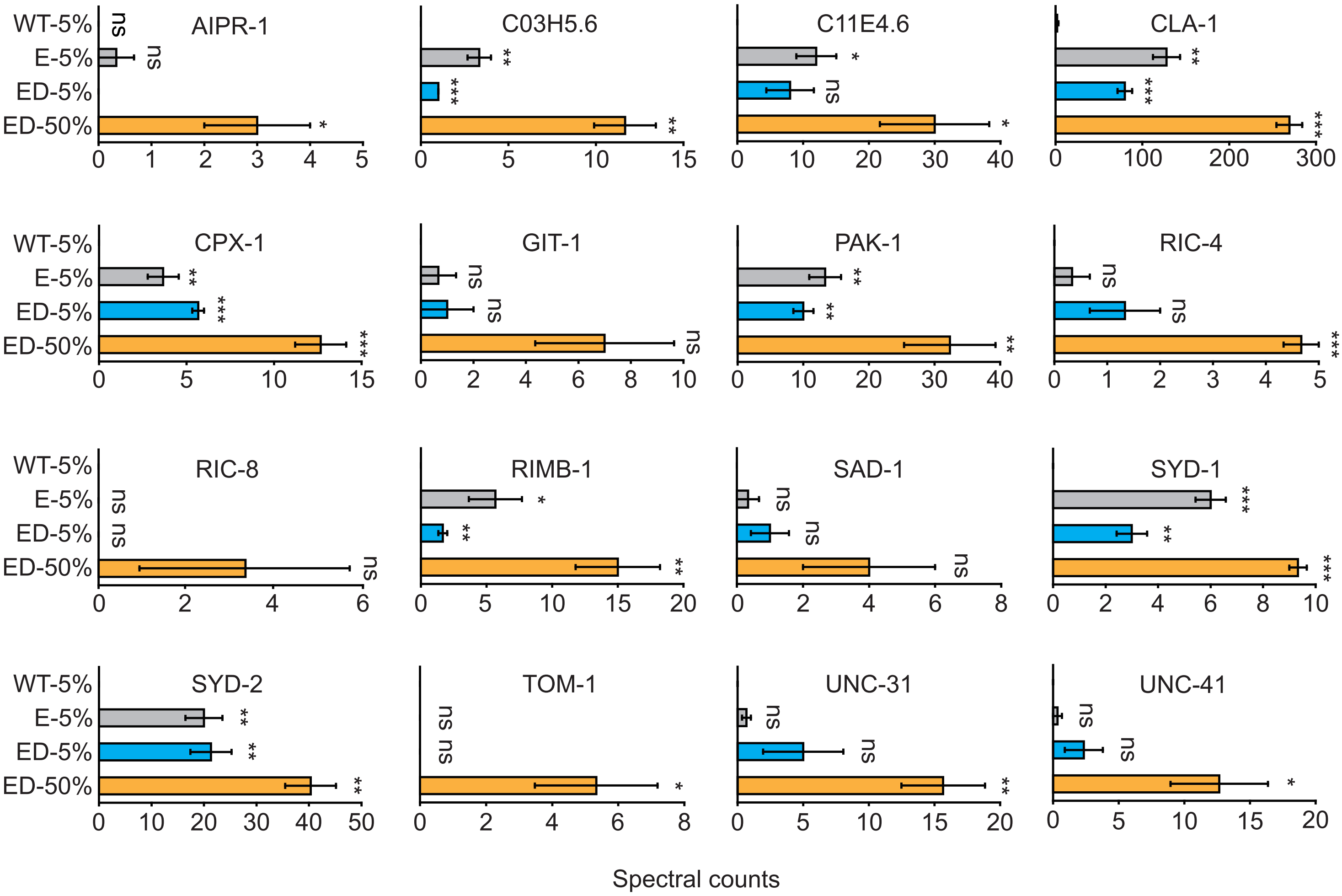

Supplement: Supplemental Figure S3 — Enrichment of synaptic proteins in depleted and undepleted samples.A, synaptic proteins in the ELKS-1 undepleted (E-5%) and depleted samples (ED-5% and ED-50%) compared to wild-type samples (WT-5%). B, mean spectral counts of synaptic proteins in wild-type, undepleted ELKS-1::TbID, and depleted 5% and 50% ELKS-1::TbID samples. p values were calculated using the unpaired Student’s t-test (two-tailed) by comparing mean spectral counts of three independent mass spec repeats of E1-5%, E2-5% or E2-50% samples with WT. Error bars represent s.e.m (ns: not significant, ∗p < 0.05, ∗∗p < 0.01, ∗∗∗p < 0.001). [file mmc3.pdf]
